# Accessibility of and barriers to the use of eye health services in Kumasi Metropolis, Ghana

**DOI:** 10.4102/phcfm.v16i1.4270

**Published:** 2024-06-25

**Authors:** Eunice A. Frempong, Diane W. van Staden

**Affiliations:** 1 Department of Optometry, Faculty of Health Sciences, University of KwaZulu-Natal, Durban, South Africa; 2 Department of Optometry, Faculty of Science, Kwame Nkrumah University of Science and Technology, Kumasi, Ghana

**Keywords:** availability, accessibility, barriers to eye health services, vision impairment, eye care services

## Abstract

**Background:**

There is a high prevalence of vision impairment and blindness in Africa. The poor access to eye health services, among other barriers, has been found to have a considerable effect on the burden of avoidable vision loss and blindness, particularly in low- and middle-income countries.

**Aim:**

To determine the accessibility of and barriers to the utilisation of eye health services in the Kumasi Metropolis of Ghana.

**Setting:**

A descriptive cross-sectional survey was conducted in the Kumasi Metropolis of the Ashanti Region in Ghana to identify barriers affecting the utilisation of eye health services.

**Methods:**

Convenience sampling was used to recruit participants visiting the eye clinics at five selected District Municipal Hospitals for the first time. Data were collected by means of questionnaires and analysed using Statistical Package for Social Sciences (SPSS).

**Results:**

Barriers faced by participants when accessing eye health services included distance to the clinic, cost of services, time spent away from work and/or school, self-medication and long waiting periods.

**Conclusion:**

The study found that eye care services in the Kumasi Metropolis, Ghana are largely accessible, but underutilised. Improvement of public health education initiatives through engagement with community groups will also enhance uptake at health care facilities.

**Contribution:**

Underutilisation of health services in the Metropolis has been identified in the study and must be addressed by health managers in various sectors. Accessibility is relatively good but can further be improved especially for the elderly to be able to utilise health care services with ease.

## Introduction

Globally, the World Health Organization (WHO) estimates that 2.2 billion people have near or distance vision impairment (VI).^1^ Of this number, 1 billion cases could have been prevented or are yet to be given the necessary attention.^1^ Most cases of blindness and VI are either avoidable or curable, yet evidence suggests that eye health services are underutilised by potential beneficiaries, even when available.^2^ Geographically, more than 90% of people affected by VI globally live in developing countries.^3^

With several global health interventions over the past 20 years, eye care services have increasingly become available to populations in developing countries. Despite this, the burden of blindness in these countries remains high.^4^ A population is said to have access to health services if they are available, and the opportunity to obtain the health services exists.^5^ The extent to which access is gained is dependent on other factors that limit utilisation of services; hence, access relies on affordability, physical accessibility and acceptability of services.^5^ Availability is also a factor that contributes to the prevention of VI worldwide, together with demographic, personal and socioeconomic factors, which may act as barriers to utilisation of the available eye health services.^6^ Availability of services varies from community to community. The availability and distribution of human and material resources for eye care are further associated with the quality of eye care delivered, including its uptake and utilisation, which all have a major effect on blindness and its prevention.^7^

Within the healthcare context, access generally relates to peoples’ ability to use health services when and where they are needed.^8^ According to Regmi and Randhawa,^8^ determinants of healthcare access include the quality of services rendered, time and distance required to access services (ease of travel), as well as the interface between service users and health providers. Poor availability and accessibility may therefore result in unmet health needs and delays in receiving the appropriate eye care. The purpose of ensuring availability and access to eye health services is to reduce avoidable vision loss and blindness without placing unreasonable financial or other burdens on the individuals or society.^9^

In Ghana, the National Health Insurance Scheme (NHIS) was introduced in 2003 to replace the user fee system. The scheme relies on premiums paid by adults employed in the informal sector who are subscribers and derives the majority of its funds from other sources for operation. Eligible individuals who are exempt from premiums include children 17 years and below, persons 70 years and above and beneficiaries of other social support programmes, which is a pro-poor intervention carried out by the central government. Health services are to be provided for free at the point of access at National Health Insurance Authority (NHIA) accredited facilities for those who have enrolled in the scheme,^10^ with coverage including all outpatient appointments, some inpatient conditions and selected drugs. For eye care, coverage includes services such as refraction, visual fields test, keratometry, cataract excision surgery and eye lid surgery.^11^

There is some evidence that members of the national health insurance enjoy increased utilisation of health services.^10^ However, eye health services such as orthoptics, optic nerve imaging, vitreo-retinal surgeries and many others that aid diagnosis and treatment of irreversibly impairing eye conditions are not factored into the scheme. This may then impose a financial burden on affected patients and become a deterrent for health service utilisation for those who cannot afford it. According to research by Alhassan et al. in 2016, the NHIS has received criticism on generosity of its benefit packages but has not been able to ensure that cardholders make no other payments while accessing health care.^12^ A national survey by the National Statistical Service (NSS) and the Ghana Health Service interviewed 13 784 people from different households. Those who visited a health care facility 6 months prior to the interview were about 30.4%, and out of this number, 45% paid fully with NHIS card, 23% a combination of the card and out-of-pocket payment, while 29% paid out-of-pocket fully.^13^ This presents the reality that the NHIS system does not guarantee zero cost and has a significant effect on affordability in health care facilities.

Several other factors may influence the use of eye health services even where they are available, affordable and accessible. Socio-economic, physical and personal barriers also affect an individual’s ability to receive the services necessary to improve vision and prevent VI.^14^ In areas where there has been a scarcity of eye health services, poor utilisation has been reported. Similar results were also recorded in a study^2^ conducted in a rural community in Nepal. Reasons for poor availability in these areas included distance to health facility, poorly developed road networks and cost of transportation.

The economic state of individuals and countries has also been shown to influence the utilisation of eye health services in various parts of the world.^4^ Non-affordability was the main contributor to low utilisation of eye health services in several rural communities in Jamaica, South Africa, Nigeria, India and Timor-Leste.^15^ Some studies specifically identified cost as an important barrier to eye health service utilisation.^16,17,18^ In Mozambique, however,^19^ cost was not seen as a significant barrier to utilisation of refractive eye health services due to the availability of low cost, ready-made spectacles. Elam and Lee^20^ conducted research on barriers to utilisation of eye care services among high-risk individuals in some communities in America. Here also, cost was the most frequently cited barrier to receiving regular eye examinations.

Hubley and Gilbert, in exploring the role of health promotion in the prevention of blindness in developing countries, investigated patients’ perspectives on barriers to the uptake of cataract services.^17^ They found one or more of the following: acceptance of impaired sight as an inevitable consequence of old age, fear of undergoing an operation, contact with individuals who had bad experiences, a lack of encouragement from family members or someone to accompany the patient to hospital, poor state of hospitals and long waiting lists, among others. In Ghana, research on barriers to utilisation of eye health facilities has been conducted in parts of the Central, Upper East and Ashanti regions.^21^ The impediments to the usage of eye health services recorded in the Upper East Region study were mainly social-related barriers such as social engagements or other priorities and ability to perform daily tasks with their condition, suggesting a change in trend over the commonly reported economic barriers like the cost of transportation and services. In the Central Region, studies reported the lack of felt need, cost and waiting time as barriers to eye health service utilisation.^22,23^ To the best of our knowledge, this study has not yet been conducted in a largely urbanised area such as the Kumasi Metropolis in the Ashanti Region of Ghana.

Good quality of eye health services^24^ and client satisfaction^15^ have been reported to enhance utilisation of eye health services. However, in the absence of an appreciation of the value of eye health services, even where good quality services exist, these may not be fully utilised unless barriers are addressed. This study was undertaken to investigate the barriers that affect utilisation of eye health services in the Ashanti Region, specifically in Kumasi, to determine whether there is a difference in barriers as compared to those identified in other studies elsewhere in Ghana and to identify possible solutions to ease any identified barriers in order to improve utilisation of eye health services and reduce the burden of avoidable blindness and VI.

## Research methods and design

The study followed a quantitative population-based cross-sectional design. It was conducted in October 2018.

### Setting

Health Services in Kumasi are organised around five (5) Sub Metro Health districts, out of which five government hospitals were selected, namely, Bantama (Suntreso Hospital), Asokwa (Kumasi South Hospital), Manhyia North (Tafo Government Hospital), Manhyia South (Manhyia Hospital) and Subin (Komfo Anokye Teaching Hospital).^25^ Each hospital was chosen due to their accessibility, availability of resources and perceived affordability as a result of the acceptance of the National Health Insurance Cards.^26^

The approximate sample size per hospital has been tabulated in Table 1.

**TABLE 1:** Approximate sample size per hospital.

Hospital	Population size per week
KATH	300
Kumasi South	200
Manhyia	200
South Suntreso	200
Tafo	240

**Total**	**1140**

KATH, Komfo Anokye Teaching Hospital.

The minimum sample size calculated was 251 patients. The study population comprised patients attending these selected district hospitals in Kumasi for the first time. Convenience sampling was used, with questionnaires administered to the participants who were available and easily accessible. Data were collected before participants were attended to by eye health personnel at the selected hospitals.

### Data collection

The primary method used to investigate accessibility to health care services and barriers that affect utilisation was a questionnaire.

The questionnaire was developed by the researcher and piloted at the University Hospital at Kwame Nkrumah University of Science and Technology in April 2018 before collecting data for the study. Changes were made during the process to include questions that will enhance and verify the usefulness of data collected to increase the likelihood of success of the study. The questionnaire comprised questions that required personal details of the respondents, their health care utilisation habits, knowledge they had about the condition(s) they had been diagnosed with, general knowledge and perceptions about the eye, the barriers that affected the utilisation of the eye health services.

Trained field workers who were intern optometrists were assigned to each of the selected hospitals to capture data within a period of 3 months, though this was not done concurrently. Data collection was supervised by trained field supervisors who were familiar with the research aim and objectives. Participants were selected from patients that visited the selected eye clinics for the first time. Questionnaires were administered by field workers via face-to-face interviews with participants using standardised questionnaires with closed and open-ended questions were used to collect data under the following themes: socio-demographic data, health care utilisation behaviour and barriers to eye health services. Convenience sampling was used to recruit participants visiting the eye clinics at five selected District Municipal Hospitals for the first time who were 10 years old and above and could speak English and/or Twi.

Data were analysed using Statistical Package for Social Sciences (SPSS) version 22.0 l. This was done after data were cleaned and validated. The primary analyses entailed descriptive analyses using frequencies, and results were presented in tables and figures.

### Ethical considerations

Ethical approval was sought from the Biomedical Research Ethics Committee of the University of KwaZulu-Natal, as well as the School of Medical Sciences/Komfo Anokye Teaching Hospital Committee on Human Research Publication and Ethics with references BE339/18 and CHRPE/AP/369/17, respectively. This research followed the ethical tenets set out in the Helsinki Declaration. Informed consent was sought from each participant before interviews. Participants were required to append a signature on hard copies of consent forms provided by the researcher either in writing or by thumbprints as proof of consent. Parents and guardians of participants below the age of 18 years were given consent forms to fill while consent forms were given or read out to children who could understand to append signature by thumbprint or writing. The socio-demographic characteristics of participants can be seen in Table 2.

**TABLE 2:** Socio-demographic characteristics of participants (*N* = 257).

Socio-demographic	Frequency (*n*)	%
**Age group (years)**		
10–19	49	19.1
20–29	104	40.5
30–39	32	12.4
40–49	21	8.2
50–59	22	8.6
60–69	16	6.2
≥ 70	13	5.0
**Gender**		
Male	103	40.1
Female	154	59.9
**Educational level**		
No formal education	15	5.8
Primary School	21	8.2
Vocational or Technical School	7	2.7
1st Degree	85	33.1
Junior High School	28	10.9
Master’s degree	11	4.3
Senior High School	76	29.6
PhD or Higher Education	1	0.4
6th Form	13	5.0
**Employment status**		
Students	30	11.6
Unemployed	87	33.9
Pensioners	9	3.5
Employed	131	51.0

## Results

### Sociodemographic characteristics

A total of 257 patients participated in the study, of which 40.1% were males and 59.9% were females. The participants’ mean age was 32.96 with a standard deviation of ±17.31, while the median of the age was 27 years within the 20–29 years age range. The age range of the participants was between 10 and 86 years with mean age of 32.9 years. A significant number of participants were within the age range 20–29 years (40.1%) while the age range with the least number of participants were 70 years and above. The majority of the participants (93%) had some form of formal education, about a third (34.7%) had a secondary level education, while 37.8% had tertiary level education. About half of the participants (51%) had some form of employment, 3.5% were pensioners who receive a retirement income from the government while the rest had no form of employment or were students in various institutions.

**FIGURE 1:**
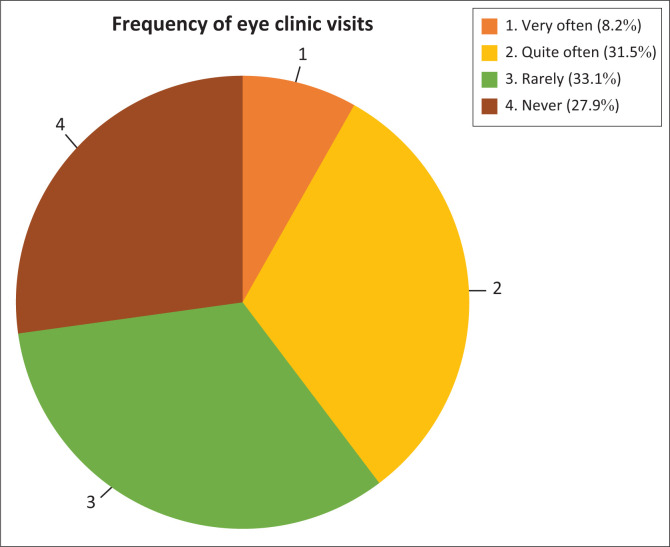
Frequency of visits.

### Frequency of visits

Most of the participants (72.8%) had visited other eye clinics before but were visiting that selected study site for the first time. One in four participants had never visited any eye clinic before (27.2%) (see Table 3).

**TABLE 3:** Report on eye health care utilisation by study participants (*N* = 257).

Variables	Frequency (*n*)	%
**Means of transportation to the clinic**		
Bus	104	40.5
Walk	89	34.6
Taxi	24	9.3
Drive	40	15.6
**Time taken to travel to clinic**		
Less than 30 min	78	30.4
30 min to 1 h	116	45.1
1 h to 2 h	50	19.5
More than 2 h	13	5.0

Barriers pertaining to accessibility impacting utilisation, which were investigated in this study, included distance or proximity to health facilities, mode of travel and travel time to the clinic. Many participants (40.5%) travelled by bus to the clinic with travel time for nearly half of them (45.1%) ranging between 30 min and 60 min.

Table 4 depicts the various barriers the study participants encountered when accessing eye health services. Some participants (27%) cited distance from their destination to the eye care centre as a barrier to accessing eye care. Regarding economic barriers, cost (medical bills, transportation) of accessing eye care recorded the highest percentage (54.1%). Some attitudinal barriers encountered when accessing health services were fear of surgical outcome (22.2%) and the self-medication practices by some participants (23.3%) who felt that they could manage their eye condition without any need for intervention.

**TABLE 4:** Barriers to utilisation of eye health services.

Barriers	Number of participants	%
**Physical**		
Too sick to travel	41	16
No escort	23	8.9
Distance	70	27.2
Transportation	40	15.7
**Economic**		
Cost (medical bills, transportation)	139	54.1
No monetary support	52	20.2
**Attitudinal**		
Can manage without intervention	38	14.9
Use of self-medication	60	23.3
Fear	57	22.2
Cultural belief	4	1.6
**Social-related**		
Time away from work and/or school	68	26.5
Time away from family	12	4.7
**Service-related**		
Long waiting time	107	41.6
Poor patient–health personnel relationship	38	14.8
Bad experiences	4	1.6
Poor state of hospital	10	3.9

With respect to the service-related barriers, 41.6% of the participants reported long waiting times before and during eye care delivery at the various eye care centres, while time away from work or school was reported by 26.5% as a social-related factor contributing to the underutilisation of eye health services.

## Discussion

This study set out to investigate the barriers affecting utilisation of eye health services in the Kumasi Metropolis of the Ashanti Region in Ghana. In general, most of the findings suggested that eye health services in the Kumasi Metropolis are available and accessible. However, these services are underutilised. Various factors have been identified as limitations to the use of eye health services in this study.

### Demographics

An important trend was identified in the age groups of the participants of this study. More than half of the participants were below the age of 30 years, with the number generally decreasing with age. Despite the assertion that the older age groups utilise health care more often than the younger groups because of the increased chances of acquiring age-related degenerative diseases as reported in some studies in Ghana and Ethiopia,^27,28^ this was not the case reported in this study. One reason for this outcome could be that elderly people tend to live in rural areas and therefore do not access health care services in the district hospitals. A study by Essuman and Mate-Kole on aging in Ghana showed that two-thirds of the elderly population live in rural settings with hardly any employment opportunities and are therefore at risk of socio-economic and health marginalisation.^29^ The authors also reported some factors that result in low utilisation of health services by the elderly being queuing frustrations, focused elderly care demands and financial burden.^29^ Another reason for this difference could be that some of the elderly are turning to private health facilities for health care. A study by Awoke et al. in 2017 showed that factors such as older age, higher education and higher income were associated with the use of private outpatient health care services.^30^ Some of the factors identified to influence the use of private health care facilities by the elderly in another study conducted in Ghana were distance and proximity, good interpersonal relationships, good quality health services, service responsiveness and logistics availability.^30^

A notable number of the participants of this study were females. This highlights the fact that females are more likely to use healthcare services as compared to their male counterparts. This may be as a result of the higher female population in the Kumasi Metropolis of the Ashanti Region. The results of a population census in 2021 showed that 51.9% of the population in the study area are females and 48.1% are males.^31^ Studies by Sekyi et al. and Geitona et al. support this finding as they found a positive influence of the female gender on utilisation of primary health care services.^28,32^

A notable link between use of eye health services and level of education was identified in this study, with the majority of the participants having a formal education, either secondary or tertiary level. There were more participants with tertiary education, followed by secondary education. This is not surprising because health care utilisation is expected to be higher among populations with higher education according to studies by Agyemang and Osei Asibey and Ye et al. They argued that people with higher educational levels are more health-conscious resulting in increased healthcare utilisation.^33,34^ This was not the case in this study where there were still high proportion of respondents who had never visited or rarely visited eye health care facilities even though many are educated. A possible rationale for this may be that higher education increases the practice of self-care. It has been indicated that self-medication is very common among tertiary students in some studies^35,36^ in which reasons such as conditions not meriting a hospital visit and long waiting time were cited.^35^

There was no significant relationship between employment and utilisation of eye health services identified in this study.

### Accessibility

Majority of participants took less than an hour to travel to the hospital, suggesting that most patients lived within reasonable distance to the hospital facility. One author^37^ described an average distance or commute of less than 30 min to 1 h to a health care facility as a measure of availability. A transport survey conducted in Ghana in 2012 found that approximately 80% of respondents from urban areas had reliable transport to health facilities to access healthcare services.^38^ A common and affordable means of transportation in Ghana are mini vans, making them an important and convenient link in the transportation system for smaller towns and villages. A significant number of respondents also indicated walking as their means of transportation. This may be an attempt to avoid long queues and traffic during morning and evening rush hours. Long queues at rush hours and acute congestion on urban roads result from low capacity of vehicles, which stems in part from poor road conditions and high maintenance costs according to research on public transport services in Kumasi.^39^

### Frequency of eye exams

Despite the relative accessibility of eye health facilities, at least half of the participants had not visited an eye clinic in more than 2 years or had never visited an eye clinic before. This indicates underutilisation of the service on the part of the population. According to the American Optometric Association, the recommended interval for comprehensive eye exams for children from age 6–17 with healthy eyes is annually. Adults from 18 to 64 years with healthy eyes are required to have their eyes tested once every 2 years, while those 65 years and older are to have annual eye exams. For those in the above age groups with eye conditions and/or those who are at risk of developing ocular conditions, an annual eye examination is recommended.^40^

The reasons for the scarce visits at eye clinics by majority of the participants could be due to cost of services, long waiting times or a lack of knowledge about the importance of having regular eye exams. In the Capricorn district of the Limpopo province in South Africa, Ntsoane et al. found a positive association between the availability and utilisation of eye health services.^41^ While utilisation was generally found to be good, it varied in relation to the proximity of eye health facilities. Studies in India have shown evidence corresponding to the result of this study that services can be available and yet have a low uptake by potential beneficiaries.^42,43^ This suggests that the problem of underutilisation of eye health services in developing countries has not been given priority.^2^

### Affordability

Affordability was a factor for more than half of the respondents in this study, emerging as one of the major barriers to utilisation of eye health services which has been cited as a major limitation to uptake of eye health services in several other studies^20,23^ as in the case of South Ethiopia where people with higher income were more likely to utilise eye health services as compared to those with lower income.^27^ This highlights the fact that, the economic state of individuals influences utilisation of eye health services. Non-affordability in another study in Timor-Leste by Palagyi et al. was also a major contributor to low utilisation of eye health services in many communities.^15^ In Mozambique, however, cost was not seen as a significant barrier to utilisation of refractive eye health services due to the availability of low cost ready-made spectacle.^19^ This suggests that where services or products are made more affordable, utilisation of eye care services should improve.

In Ghana, the Health Insurance coverage improves eye care-seeking behaviour of individuals who make use of it by reducing the cost of health care in some government and non-government eye health facilities, because it covers all outpatient admissions. It also covers services such as refraction, cataract removal, eye lid surgery, keratometry and visual field test.^10^ However, some necessities such as most drugs prescribed at the health facilities, spectacles and low vision aids are not covered and have to be paid out-of-pocket by individuals who need them. Also, more specialised services such as orthoptic care, glaucoma diagnostics that require optic nerve imaging, gonioscopy, vitreo-retinal surgeries, laser surgeries and many other interventions to prevent blindness and VI fall outside of this coverage, thus increasing the cost of accessing certain levels of eye care considerably.

### Other barriers

An attitudinal barrier identified in this study was fear, a concern among almost a quarter of participants. Fear has been reported in other studies^23,22^ as a non-significant barrier in other parts of Ghana. Cataract surgical uptake among older adults in Ashanti Region, which has one of the highest levels of self-reported cataract cases, was found to be lower than other regions.^44^ Ocansey et al.^23^ identified the main reason for poor uptake of eye health services as being an attitude towards the services as a result of the perception that eye conditions were minor and not important enough to access health services.^23^ Social attitude among the Indian population living in West London was that visual health was reported as not being a priority, as the elderly are resigned to a ‘fate’ of poor vision in old age.

Another identified effect on utilisation in eye health facilities was self-treatment. Participants preferred to purchase over-the-counter drugs in private pharmacies or use left-over medications previously given at the hospital to them or their relatives and friends to relieve symptoms they were feeling. Self-medication is known to be very common in developed and developing nations.^35,36,45,46^ Irresponsible self-medication practices can have harmful consequences such as increased risk of drug abuse and adverse drug effects and results in reduced clinic visits.^45^

The social-related barrier identified in this research was time away from work or school. A study by Merepa et al. in the Upper East Region of Ghana reported a predominance of sociallyrelated factors over service-related factors.^21^ Merepa et al., however, indicated that factors such as availability and accessibility, which have been major barriers to eye health service utilisation in previous studies^6^ have seen a change in trend with time and so were not significant barriers in their findings.^21^ Their research showed the major barriers affecting utilisation of eye health services were priority for school, work and other engagements and ability to perform daily tasks with eye conditions. This trend is seen also in the Ashanti Region according to the findings of this study.^21^

Time away from work and school was identified as a cause of some patients’ inability to utilise eye health services. When the underlying eye condition did not cause pain and discomfort, patients tended to consider it as unnecessary to seek attention.^47^ This avoidance could later lead to VI or blindness if not addressed.

A major deterrent to eye care utilisation identified in this study was the long waiting time at eye clinics. Waiting time refers to the overall time taken by a patient from the time of arrival to the time spent at each service point until the last service is rendered.^48^ In Ghana, respondents of a study conducted in the Central Region^22^ reported similar results whereby waiting time was a major barrier to eye care utilisation, second to cost. On the contrary, findings from a study conducted in the Eastern Region of Ghana showed that waiting time, though a barrier, did not have a significant effect on utilisation. The reason for long waiting time reported as a barrier among respondents could potentially be high population density in the affected areas. At the time of the study, the Ashanti Region had the highest estimated population at 5 552 177^49^ as compared to the other regions, including the Greater Accra Region, which has the highest number of health personnel and has a population of 4 738 205.^49^

Another cause of long waiting time could be the early arrival of patients long before the clinic starts operating. Muzinguzi^48^ reported that the majority of patients in an outpatient department of a hospital in Uganda arrived between 07:00 and 10:00 but had to wait for an average of about 4–5 h to see the doctor or receive treatment. In addition, patients who are referred for further investigations return to either join the queue of newly arrived patients, or newly arrived patients have to wait for those returning with investigation results to see the doctors again. This results in extended wait times and service delays. Inadequate registration and dispensing staff also result in long waiting periods.^48^

Behaviours that undermine safety and trust within the hospital threaten patient outcomes.^50^ A study on factors that influence receipt of eye care conducted in United States reported that participants felt their primary health providers did not communicate information with them about eyesight, nor did they conduct basic eye screenings.^51^ One of the most frequently cited barriers to utilisation in a study^20^ was patient–doctor relationship. Poor patient-health personnel relationship was also identified as a service-related barrier in this study similar to the findings of Elam et al.^20^ and Patel et al.^52^ Poor relationship and communication could be due to high patient numbers that afford doctors limited time to communicate extensively about each person’s eye condition.

### Limitations

The findings of this study were self-reported and hence could contain information bias or recall bias. Furthermore, the findings may not applicable to the whole country and authors recommend further and broader studies be conducted to obtain a complete picture of the problem. Another limitation is that the study was conducted in one out of the 43 district assemblies in the Region due to the lack of logistical and financial resources and its outcome may not be reflective of the other districts or the whole Region.

## Conclusion

While the availability and accessibility of eye health services in the Kumasi Metropolis appear adequate, utilisation remains a concern. There was low utilisation of services among the elderly as compared to the younger age groups. Females were more likely to use eye health care services in contrast with their male counterparts. Education did not have a positive effect on utilisation of services in this study as it increases the practice of self-care and self-medication among the highly educated. Accessibility to eye health care services was relatively reasonable for those living in the Kumasi Metropolis. The frequency of eye clinic visits was poor though services were accessible indicating underutilisation among members. Cost was the most significant barrier to utilisation in spite of the use of the NHIS cards, suggesting the need for an improved National Health Insurance system coverage to include more essential products and services such as spectacles, low vision aids, orthoptic treatment and essential imaging tests needed for early diagnosis of vision-impairing eye diseases. Fear of being diagnosed of a condition demanding surgery was also a remarkable attitudinal barrier highlighted in this study. Self-treatment was found to be associated with underutilisation of eye health services in the Metropolis. Time away from work and school was the major social-related barrier affecting the use of ophthalmic services while long waiting times and poor doctor–patient relationships were service-related barriers affecting utilisation.

## References

[1] WHO. Blindness and visual impairment [homepage on the Internet]. 2021 [cited 2021 Oct 21]. Available from: https://www.who.int/news-room/fact-sheets/detail/blindness-and-visual-impairment

[2] Gnyawali S, Bhattarai D, Mp U. Utilization of primary eye health services by people from a rural community of Nepal. Nepal J Ophthalmol. 2012;4(1):96–101. 10.3126/nepjoph.v4i1.5859 22344005

[3] World Health Organisation (WHO). World report on vision [homepage on the Internet]. 2019 [cited 2019 Dec 15]. Available from: https://apps.who.int/iris/bitstream/handle/10665/328717/9789241516570-eng.pdf

[4] Burton M, Ramke J, Marques A, et al. The Lancet Global Health Commission on global eye health: Vision beyond 2020. Lancet Glob Health. 2021;9(4):489–551. 10.1016/S2214-109X(20)30488-5 PMC7966694 33607016

[5] Gulliford M, Figueroa-Munoz J, Morgan M, Hudson M. What does ‘access to health care’ mean? J Health Serv Res Policy. 2002;7(3):186–188. 10.1258/135581902760082517 12171751

[6] Ntsoane M, Oduntan O. A review of factors influencing the utilization of eye care services. Afr Vis Eye Health. 2010 Dec;69(4):182–192. 10.4102/aveh.v69i4.143

[7] Prozesky D. Advocacy for eye health. Community Eye Health J. 2007;20(64):57–59. PMC2206315 18330435

[8] Regmi K, Randhawa G. Access to health care: Issues of measure and method. Prim Health Care. 2013;3:136.

[9] World Health Organisation (WHO). Universal Health Coverage (UHC); 2022 [cited 2023 May 03]. Available from: https://www.who.int/news-room/fact-sheets/detail/universal-health-coverage-(uhc)

[10] Potter A, Debrah O, Ashun J, Blanchet K. Eye Health Systems Assessment (EHSA). Accra: Ghana country report, sight savers, International Centre for Eye Health; 2013.

[11] NHIS. NHIS benefits package [homepage on the Internet]. 2021 [cited 2023 Oct 14]. Available from: https://www.nhis.gov.gh/benefits.aspx

[12] Alhassan RK, Nketiah-Amponsah E, Arhinful DK. A review of the national health insurance scheme in Ghana: What are the sustainability threats and prospects? PLoS One. 2016 Nov 1;11(11):e0165151. 10.1371/journal.pone.0165151 27832082 PMC5104458

[13] Haw NJL. Utilization of the Ghana National Health Insurance Scheme and its association with patient perceptions on healthcare quality. Int J Qual Health Care. 2019;31:485–491. 10.1093/intqhc/mzy185 30165414

[14] Kumari R, Singh K, Dubey G, et al. Chronic impediment in utilization of eye-care services. J Ophthalmol Res. 2020;3:45–56. 10.26502/fjor.2644-00240020

[15] Palagyi A, Ramke J, Du Toit R, Brian G. Eye care in Timor-Leste: A population-based study of utilization and barriers. Clin Exp Ophthalmol. 2008 Jan;36(1):47–53. 10.1111/j.1442-9071.2007.01645.x 18190597

[16] Balo P, Serouis G, Banla M, Agla K, Djagnikpo P, Gué K. Knowledge, attitudes and practices regarding glaucoma in the urban and suburban population of Lomé (Togo). Sante [serial online]. 2004 [cited 2021 Feb 07];14(3):187–191. Available from: https://pubmed.ncbi.nlm.nih.gov/15563419/15563419

[17] Hubley J, Gilbert C. Eye health promotion and the prevention of blindness in developing countries: Critical issues. Br J Ophthalmol. 2006 Mar;90(3):279–284. 10.1136/bjo.2005.078451 16488944 PMC1856969

[18] Ndegwa L, Karimurio J, Okelo RO, Adala HS. Barriers to utilisation of eye care services in Kibera slums of Nairobi. East Afr Med J. 2005 Oct;82(10):506–508. 10.4314/eamj.v82i10.9347 16450677

[19] Thompson S, Naidoo K, Gonzalez-Alvarez C, Harris G, Chinanayi F, Loughman J. Barriers to use of refractive services in Mozambique. Optom Vis Sci. 2015 Jan 1;92(1):59–69. 10.1097/OPX.0000000000000431 25379630 PMC4274338

[20] Elam A, Lee PP. Barriers to and suggestions on improving utilization of eye care in high-risk individuals: Focus group results. Int Sch Res Notices. 2014;2014:527831. 10.1155/2014/527831 27379302 PMC4897391

[21] Merepa S, Akowuah P, Abazele A, et al. Barriers to utilization of eye care services in the Upper East Region, Ghana. Adv Ophthalmol Vis Syst. 2017;7(6):00240. 10.15406/aovs.2017.07.00240

[22] Ilechie A, Otchere H, Darko-Takyi C, Abraham CH. Access to and utilization of eye care services in Ghana. Int J Health Res. 2013;6(3):7–15.

[23] Ocansey S, Kyei S, Gyedu B, Awuah A. Eye care seeking behaviour: A study of the people of Cape Coast Metropolis of Ghana. J Behav Health Serv Res. 2014;3(2):101. 10.5455/jbh.20140219014308

[24] Chandrashekhar TS, Bhat HV, Pai RP, Nair SK. Coverage, utilization and barriers to cataract surgical services in rural South India: Results from a population-based study. Public Health. 2007 Feb 1;121(2):130–136. 10.1016/j.puhe.2006.07.027 17215012

[25] Ghana Statistical Service (GSS). 2010 Population and Housing Census. District analytical report, Kumasi Metropolitan Assembly. Accra: Ghana Health Service; 2014.

[26] Kumasi Metropolitan Assembly (KMA). KMA medium term development plan 2010–2013. Kumasi: Kumasi Metropolitan Assembly, Ministry of Local Government, Rural Development and Environment; 2011.

[27] Morka E, Yibekal B, Tegegne M. Eye care service utilization and associated factors among older adults in Hawassa city, South Ethiopia. PLoS One. 2020;15(4):e0231616. 10.1371/journal.pone.0231616 32298344 PMC7162464

[28] Sekyi S, Laari B, Kwaku G, Ampofo M. Heterogeneous effects of demographic factors on healthcare utilisation in Ghana. Ghana J Dev Stud. 2023;20(1):82–97. 10.4314/gjds.v20i1.5

[29] Essuman A, Mate-Kole CC. Ageing in Ghana. In: Selin H, editor. Aging Across Cultures: Growing Old in the Non-Western World. Springer Verlag, 2021; pp. 1–11.

[30] Awoke MA, Negin J, Moller J, et al. Predictors of public and private healthcare utilization and associated health system responsiveness among older adults in Ghana. Glob Health Action. 2017;10(1):1301723. 10.1080/16549716.2017.1301723 28578615 PMC5496095

[31] Ghana Statistical Service. Ghana 2021 population and housing census general report volume 3A. Accra: Ghana Statistical Service; 2021.

[32] Geitona M, Zavras D, Kyriopoulos J. Determinants of healthcare utilization in Greece: Implications for decision-making. Eur J Gen Pract. 2007;13(3):144–150. 10.1080/13814780701541340 17853172

[33] Ye L, Shia BC, Fang Y, Lee TS. Heterogeneous health profiles and healthcare utilization of the middle-aged and elderly with multiple health insurance schemes in China. Public Health. 2019 May 1;170:61–69. 10.1016/j.puhe.2019.01.011 30954778

[34] Agyemang S, Asibey BO. Effect of education on health care utilization in rural Ghana: The case of selected communities in the Bekwai Municipality. KNUST J Geogr Dev [serial online]. 2018;2(1). [cited 2021 Aug 10]. Available from: https://www.researchgate.net/publication/327023818

[35] Gbadago C, Stephens J, Calys-Tagoe B, Kenu E. Factors influencing self-medication among students of University of Ghana, Legon. Postgrad Med J Ghana. 2021;10(1):22–30. 10.60014/pmjg.v10i1.247

[36] Kasulkar AA, Gupta M. Self-medication practices among medical students of a private institution. Indian J Pharm Sci. 2015;77(2):178–182. https://doi.org/10.4103%2F0250-474x.156569 26009650 10.4103/0250-474x.156569 PMC4442466

[37] Kuhlthau K. Measures of availability of health care services for children. Acad Pediatr. 2011;11:42–48. 10.1016/j.acap.2010.11.007 21570016

[38] Takyi H, Poku K, Anim K. An assessment of traffic congestion and its effect on productivity in urban Ghana. Int J Bus Soc Sci. 2020;4(3):225–234.

[39] Poku-Boansi M, Adarkwa K. The determinants of demand for public transport services in Kumasi, Ghana. J Sci Technol. 2014;33(3):60. 10.4314/just.v33i3.7

[40] American Optometric Association. Comprehensive eye exams [homepage on the Internet]. 2023 [cited 2024 Jan 09]. Available from: https://www.aoa.org>healthyeyes>caring-for-your-eyes

[41] Ntsoane M, Oduntan O, Mpolokeng B. Utilisation of public eye care services by the rural community residents in the Capricorn district, Limpopo Province, South Africa. Afr J Prim Health Care Fam Med. 2012 Feb;4(1):1–7. 10.4102/phcfm.v4i1.412

[42] Robin AL, Nirmalan PK, Krishnadas R, et al. The utilization of eye care services by persons with glaucoma in rural south India. Trans Am Ophthalmol Soc. 2004;102:45–47. 10.1167/iovs.04-0285 PMC1280086 15747744

[43] Bhagwan J, Rastogi IM, Malik JS, et al. Knowledge, attitude and practices regarding cataract surgery among senile cataract cases in Haryana. Indian J Community Med. 2006;31(2):66–68.

[44] Ackuoko-Dogbe E, Yawson A, Biritwum R. Cataract surgical uptake among older adults in Ghana. Ghana Med J. 2015;49(2):84–89. 10.4314/gmj.v49i2.4 26339091 PMC4549822

[45] Zheng Z, Feng Z, Zhang D, et al. Does self-medication reduce medical expenditure among the middle-aged and elderly population? A four-wave longitudinal study in China. Front Public Health. 2022;10:1047710. 10.3389/fpubh.2022.1047710 36711405 PMC9874163

[46] Alghanim SA. Self- medication practices among patients in a public health care system. East Mediterr Health J. 2011;17(5):409–416. 10.26719/2011.17.5.409 21796954

[47] Elliot A, Chou C, Zhang X, Al E. Eye care utilization among women aged 40 years with eye diseases in 19 states (2006–2008). MMWR Morb Mortal Wkly Rep. 2010;59(19):588–591. 20489682

[48] Muzinguzi C. Patient waiting time and associated factors at the Assessment Center, General out-patient Department, Mulago Hospital Uganda [Unpublished Dissertation] [homepage on the Internet]. Makerere: Makerere University; 2013 [cited 2020 Feb 02]. Available from: http://www.researchgate.net

[49] Ghana Health Service (GHS). Ghana Health Service report 2016. The health sector in Ghana. Facts and figures 2017. Accra: Ghana Health Service; 2018.

[50] Layne D, Nemeth L, Mueller M, Martin M. Negative behaviors among health care professionals: Relationship with patient safety culture. Healthcare. 2019;7(1):23. 10.3390/healthcare7010023 30717313 PMC6473815

[51] Alexander RJ, Miller N, Cotch M, Janiszewski R. Factors that influence receipt of eye care. Am J Health Behav. 2008;32(5):547–556. 10.5993/AJHB.32.5.10 18241139 PMC2941200

[52] Patel D, Baker H, Murdoch I. Barriers to uptake of eye care services by the Indian population living in Ealing, west London. Health Educ J. 2006 Sep;65(3):267–276. 10.1177/0017896906067777

[53] Frempong EA. Accessibility of and barriers to the use of eye health services in Kumasi Metropolis, Ghana [unpublished dissertation]. University of KwaZulu-Natal, South Africa; 2022. 10.4102/phcfm.v16i1.4270 PMC11219546 38949439

